# Direct Visualization of the Charge Transfer in a Graphene/α-RuCl_3_ Heterostructure via Angle-Resolved Photoemission Spectroscopy

**DOI:** 10.1021/acs.nanolett.3c01974

**Published:** 2023-08-28

**Authors:** Antonio Rossi, Cameron Johnson, Jesse Balgley, John C. Thomas, Luca Francaviglia, Riccardo Dettori, Andreas K. Schmid, Kenji Watanabe, Takashi Taniguchi, Matthew Cothrine, David G. Mandrus, Chris Jozwiak, Aaron Bostwick, Erik A. Henriksen, Alexander Weber-Bargioni, Eli Rotenberg

**Affiliations:** † Advanced Light Source, Lawrence Berkeley National Laboratory, Berkeley, California 94720, United States; ‡ The Molecular Foundry, Lawrence Berkeley National Laboratory, Berkeley, California 94720, United States; § Center for Nanotechnology Innovation @ NEST, Istituto Italiano di Tecnologia, Pisa 56127, Italy; ∥ Department of Physics and Institute for Materials Science and Engineering, Washington University in St. Louis, St. Louis, Missouri 63130, United States; ⊥ Physical and Life Sciences Directorate, Lawrence Livermore National Laboratory, Livermore, California 94550, United States; # Research Center for Functional Materials, National Institute for Materials Science, 1-1 Namiki, Tsukuba 305-0044, Japan; ¶ International Center for Materials Nanoarchitectonics, National Institute for Materials Science, 1-1 Namiki, Tsukuba 305-0044, Japan; ■ Material Science & Technology Division, Oak Ridge National Laboratory, Oak Ridge, Tennessee 37831, United States

**Keywords:** Graphene, a-RuCl_3_, p−n junction, electronic structure, angle-resolved photoemission spectroscopy, low energy electron microscopy

## Abstract

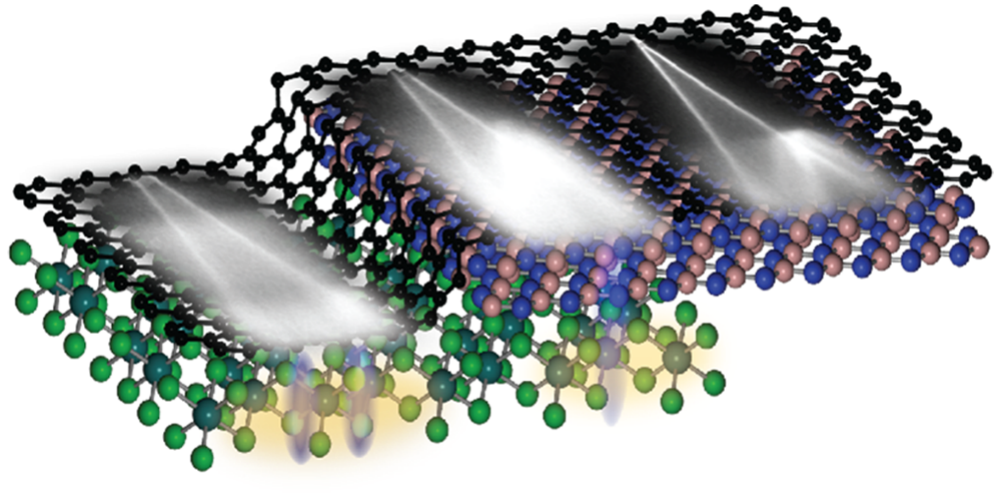

We investigate the electronic properties of a graphene and α-ruthenium trichloride (α-RuCl_3_) heterostructure using a combination of experimental techniques. α-RuCl_3_ is a Mott insulator and a Kitaev material. Its combination with graphene has gained increasing attention due to its potential applicability in novel optoelectronic devices. By using a combination of spatially resolved photoemission spectroscopy and low-energy electron microscopy, we are able to provide a direct visualization of the massive charge transfer from graphene to α-RuCl_3_, which can modify the electronic properties of both materials, leading to novel electronic phenomena at their interface. A measurement of the spatially resolved work function allows for a direct estimate of the interface dipole between graphene and α-RuCl_3_. Their strong coupling could lead to new ways of manipulating electronic properties of a two-dimensional heterojunction. Understanding the electronic properties of this structure is pivotal for designing next generation low-power optoelectronics devices.

In recent years, there has been a surge in interest in heterostructures composed of different two-dimensional (2D) materials.^1−3^ These systems offer unique electronic properties that arise from their interfacial interactions, making them promising candidates for novel electronic and optoelectronic devices.^4,5^ The absence of a covalent chemical bond between the layers opens the route toward designing 2D quantum systems that hold the promise to unlock the post-Moore era.^6,7^ One particularly exciting development is the recent discovery of permanent charge transfer induced in graphene by proximity to α-RuCl_3_ (RuCl_3_ hereafter). In turn, this offers a route to explore the physics of charge-doped Mott insulators.^8−12^

RuCl_3_ is a layered transition metal compound with a honeycomb lattice structure similar to graphene. However, unlike graphene, it is a Mott material with insulating behavior arising from strong electronic correlations.^13^ At low temperatures, the complex competition of magnetic interactions ultimately stabilizes a zigzag antiferromagnet in RuCl_3_.^14^ However, the influence of doping, for instance by photoinduced charged carriers, is predicted to stabilize ferromagnetic order.^15^ Additionally, RuCl_3_ is classified as a Kitaev material due to its strong spin–orbit coupling, crystal field, and electronic correlations, which lead to anisotropic exchange interactions that favor the formation of a quantum spin liquid expected to host Majorana fermions.^16−20^ These quasiparticles have non-Abelian statistics and are essential for topological quantum computation.^21^ Nonetheless, the evidence for such behavior in RuCl_3_ is still under debate^22^ and seems to be strongly affected by the presence of crystal defects, which promote impurity scattering and non-Kitaev interactions.^23^

When graphene is brought in contact with RuCl_3_, a charge transfer occurs between the two materials due to their different work functions and electronic structures.^8^ This charge transfer can modify and hybridize the electronic properties of both materials^10^ as well as influence the magnetism in RuCl_3_.^15,24^ The coupling between graphene and RuCl_3_ can modify the electronic band structure of RuCl_3_ and enhance its spin–orbit coupling, potentially impacting the Kitaev physics in the material.^9,24^ Anomalous quantum oscillations have been reported in the Gr/RuCl_3_ heterostructure and explained within the Kitaev-Kondo lattice model.^25−27^ The strong charge transfer has also been used to create modulation-doped graphene where a lateral thickness variation of a tunnel barrier changes the magnitude of the charge transfer between graphene and RuCl_3_,^11^ enabling ultrasharp (less than 5 nm wide) p–n junctions,^12^ which were also observed in nanobubbles of graphene on RuCl_3_.^28^ The interaction between graphene and RuCl_3_ can also lead to plasmon polaritons at the interface.^29^ The coupling between plasmon polaritons and the Mott physics in RuCl_3_ could unlock new ways of manipulating light and electronic properties, with potential applications in sensing, imaging, and communication. Moreover, by leveraging the unique passive doping control (no gating needed) of RuCl_3_ over graphene, we envision the creation of low-power devices that exhibit enhanced light-harvesting capabilities and precise control over optical signals.^30^

Here, we employ a combination of experimental techniques to better investigate the electronic properties of the interface between RuCl_3_ and graphene. Nanometer-scale spatially resolved photoemission spectroscopy (nanoXPS) and low-energy electron microscopy (LEEM) are used to explore the electronic properties of the heterostructure, allowing for a direct visualization of its charge transfer.

It is possible to effectively map the core levels of 2D systems and the dispersive electronic band structure of the heterostructure with submicron spatial resolution via nanoXPS and angle-resolved photoemission spectroscopy (nanoARPES).^31^ LEEM allows imaging of the morphology and electronic properties of heterostructures with high spatial resolution. By using low-energy electrons to probe the surface of the material, we can investigate local variations in electronic properties to study their evolution over time. By comparing our experimental results with the calculations present in the literature, we validate our findings and provide a more complete understanding of the electronic properties of the heterostructure.

The experimental data show a massive charge transfer from graphene to RuCl_3_, clearly visible in the nanoARPES data and reflected in the core levels measured via nanoXPS. LEEM analysis provides a value of the shift in work function that is much lower than the band shift measured via nanoARPES, consequent to the charge transfer between the layers. This discrepancy can be attributed to the presence of a dipole at the interface that greatly affects the work function value.^32^ Moreover, the appearance of a band below the Fermi level, not observed in other ARPES experiments, can be attributed to the effect of charge transfer on RuCl_3_. The most straightforward interpretation suggests that electrons from graphene have partially occupied the typically unoccupied upper Hubbard band, causing it to shift below the Fermi level. Alternatively, in a study of adatom-doped RuCl_3_, Zhou et al. identified the appearance of such a band between the lower Hubbard band and Fermi level as an unconventional Mott transition driven by the charge transfer.^33^ Either way, this new spectral weight shows that charge transfer from graphene to RuCl_3_ induces significant changes in the electronic structure of the system. The presence of this band provides further evidence of the complex electronic interactions occurring at the interface and highlights the role of charge transfer in driving unconventional electronic phenomena in this system.

We fabricated a heterostructure composed of a thick hexagonal boron nitride (h-BN) substrate, with three other materials exfoliated on top: graphene, thin h-BN (2 nm), and RuCl_3_ (Figure 1(a)). The fabrication and experimental details are reported in the Supporting Information. The device has three distinct regions: one with graphene on thick h-BN as a reference (Gr/h-BN), one with graphene directly on RuCl_3_ (Gr/RuCl_3_), and one with a thin h-BN flake sandwiched between graphene and RuCl_3_ (Gr/h-BN/RuCl_3_). The thin h-BN acts as a buffer to decrease the Gr-RuCl_3_ interactions.^11,28^ The thick h-BN substrate provides a stable and flat surface for other materials and minimizes the effect of the underlying substrate on the electronic properties of the heterostructure. A sketch of the three regions is reported in Figure 1(b) with a coherent color scheme. Figure 1(c) displays the heterostructure contour, where the contrast is given by the counts of the photoelectrons coming from the valence band of RuCl_3_ at a binding energy of −1.3 eV. The contrast allows for identifying the three regions described above. The color scheme for the three colored squares on the map is consistent with the sketch in panel (b) and confirmed by the core level analysis via XPS. We focus on the peaks originating from Cl, Ru, and C core levels, reported in Figure 1(d,e). The most informative peak regarding the location of the RuCl_3_ region is the Cl 2p core level. Its signal decreases when the h-BN buffer is present and disappears entirely outside the RuCl_3_ flake. The Ru 3d_3/2_ core level partially overlaps with that of C 1s. It is possible to fit and track the evolution of the C 1s peak for the three different regions (Figure 1(f)). The fitting is performed considering one Doniach-Sunjic (DS) asymmetric line shape^34^ for graphene and one DS for Ru 3d_3/2_, plus a Gaussian peak to take into account the broad and weak contribution from the 3d_5/2_ peak. While the Ru level remains roughly at fixed position, the C peak progressively shifts toward lower binding energy when increasing the coupling strength between graphene and RuCl_3_. An overall shift of about −750 meV is observed for C 1s from the Gr/h-BN region. RuCl_3_ is expected to induce a significant electron depletion in graphene^8,9^ that is reflected on an electrostatic shift of the C core levels and the whole graphene band structure.

**Figure 1:**
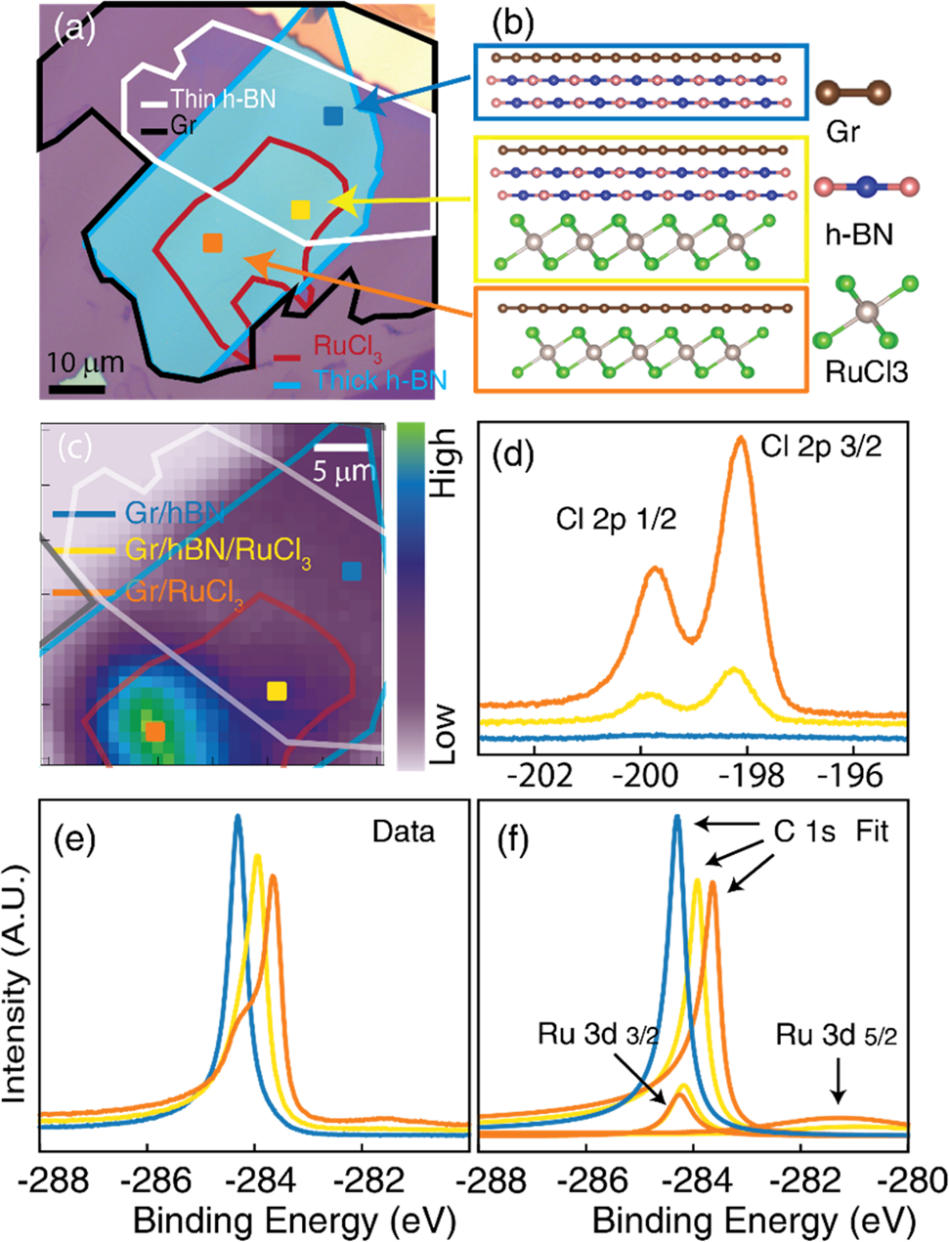
(a) Optical image of the analyzed device. False-color contours are used to highlight the different layers. (b) Sketch of the side view of three regions of interest. RuCl_3_ must be considered as multiple layers, even though one layer is depicted for neatness. (c) Photoelectron intensity map collected at *E* – *E*_*F*_ = −1.3 eV. The colored profiles match the flakes highlighted in panel (a). (d) The Cl 2p core level collected in the regions highlighted in panel (c) with the corresponding color scheme. (e) Ru 3d and C 1s core levels collected from the same points of panel (d). (f) Fit of the Ru 3d and C 1s level for the data reported in panel (e).

To directly visualize the electronic properties of the system, we conducted a nanoARPES study. This study enabled us to observe the electronic band structure in three specific regions highlighted in Figure 1(c). In Figure 2(a), we present the bands of the Gr/h-BN region, which are close to the neutrality point. In Figure 2(b,c), we show the band structures of the Gr/h-BN/RuCl_3_ and Gr/RuCl_3_ regions, respectively. Notably, the graphene bands in these regions are shifted upward by approximately 500 meV where the h-BN layers separate the graphene and RuCl_3_ crystals and by 750 meV where RuCl_3_ is in direct contact with graphene, indicating a progressive p-doping when reducing the distance between graphene and RuCl_3_. The charge transfer from graphene to RuCl_3_ is responsible for the shift of the Dirac cone and is similarly manifested in the position of the C 1s core level, where the observed chemical shift, as illustrated in Figure 1, can be also ascribed to the presence of an electrostatic dipole effect.^35^ The influence of charge transfer and the resulting interface dipole is evident in the upward shift of the h-BN bands (indicated by white arrows) by approximately 1 eV, relative to the region without RuCl_3_ as shown in Figure 2(a).

**Figure 2:**
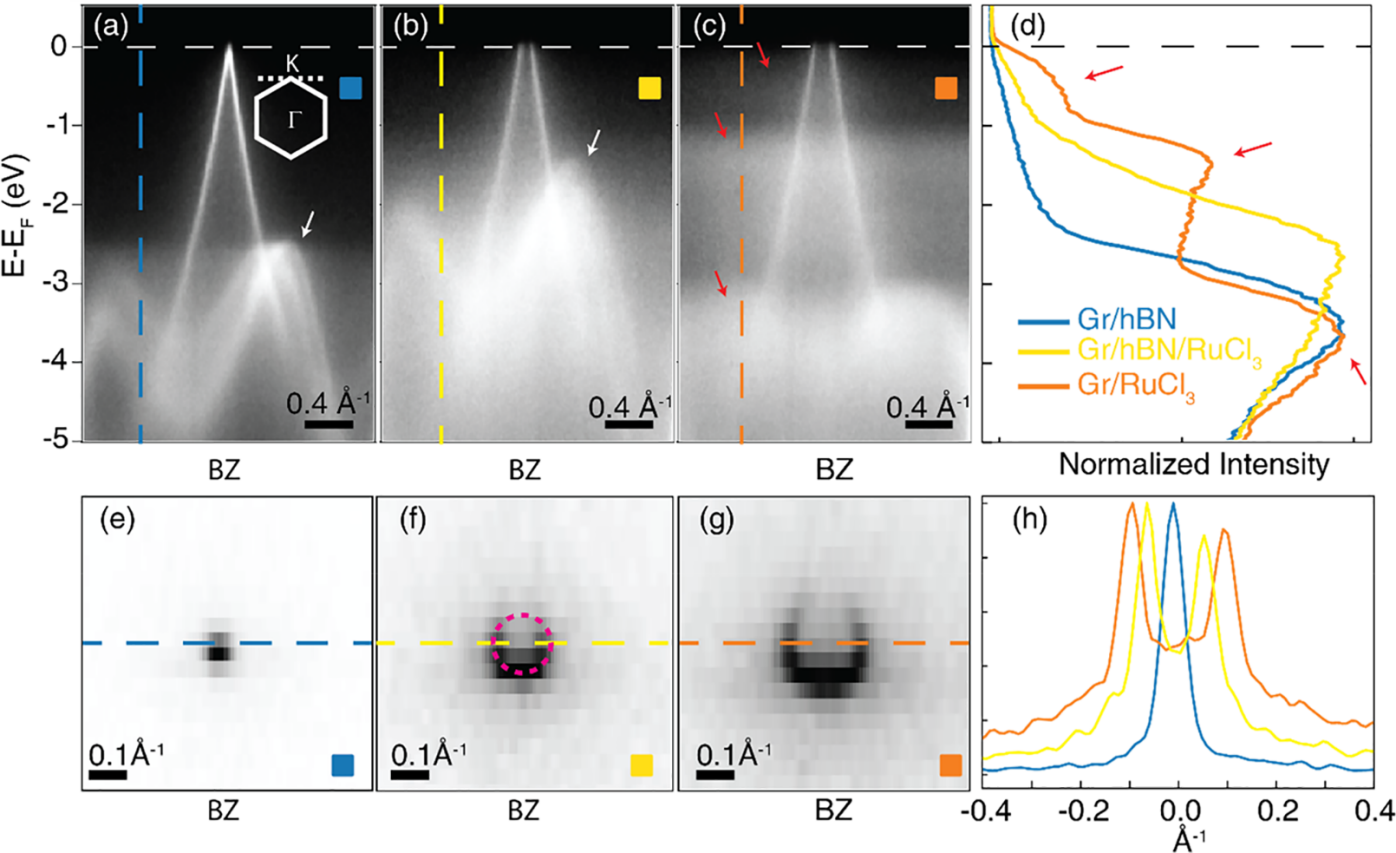
(a–c) Band structure collected around graphene K (point as depicted by panel (a) inset) from the three regions described above with a consistent color scheme. The horizontal dashed line is the Fermi level. The white arrows highlight the h-BN bands, the red arrows the RuCl_3_ bands. (d) EDCs collected from panels (a–c) along the vertical dashed line. The red arrows highlight the corresponding states in panel (c). (e–g) Fermi surface of the band structure from the sample regions with the corresponding color scheme. The dashed red circle in panel (f) approximates the graphene Fermi surface. (h) MDCs collected across the dashed lines in panels (e–g).

Because of the short mean free path of the photoelectrons, the RuCl_3_ bands are visible only in the region where graphene is in direct contact, with no intervening h-BN buffer. The RuCl_3_ bands are identified by studying the energy distribution curves (EDCs) taken along the dashed line in panels (a–c) of Figure 2 (Figure 2(d)). The RuCl_3_ electronic structure, highlighted by three red arrows, displays two dispersionless bands centered at binding energies of ∼0.5 and ∼1.3 eV and a third more dispersive band at a deeper binding energy (∼3.8 eV).

The band centered at −1.3 eV is very likely to correspond to the lower Hubbard band, as indicated by Biswas et al.^9^ However, it is worth noting that the observed energy position of the lower Hubbard band may appear to be lower than that predicted by computational models. This discrepancy could be attributed to the challenges in accurately estimating the energy gap using, e.g., density functional theory (DFT) calculations. Factors such as electron–electron interactions and correlation effects, which are not fully captured in DFT calculations, can influence the energy position of the lower Hubbard band.

Regarding the band at −3.8 eV, it is identified as an in-plane orbital and labeled as Cl p bands, originating from the Cl orbitals within the RuCl_3_ structure.^33^

The presence of the band with spectral weight centered at about 0.5 eV below the Fermi level, which is typically not observed in ARPES experiments conducted on bulk RuCl_3_,^33,36^ suggests that its emergence is a result of the interaction between RuCl_3_ and graphene. This band can be understood as the upper Hubbard band, which is typically unoccupied, being partially filled by electrons transferred from graphene and consequently shifted below the Fermi level. Another explanation put forth by Zhou et al. proposes that the introduction of dopants on the surface of RuCl_3_ leads to the population of new bands near the Fermi level.^33^ These bands are attributed to an unconventional Mott transition, as described by the authors. It is possible that RuCl_3_ undergoes a similar Mott transition when in contact with graphene, as observed in the case of Rb and K doping in ref (33).

A more quantitative estimate of the total amount of charge transferred between the layers, with and without the h-BN buffer layer, is given by considering the Fermi surface for each of the three regions, as reported in Figure 2(e–g). The Fermi surface of graphene exhibits a characteristic area of reduced intensity known as the “dark corridor”. This phenomenon arises due to the interference of photoelectrons that are emitted from the two identical carbon atoms within each unit cell of graphene’s honeycomb lattice.^37^ The momentum distribution curves (MDCs) collected along the dashed lines on the Fermi surface are displayed in Figure 2(h). By fitting with two Lorentzian curves, the position of their maxima is used to evaluate the Fermi surface area, approximated as a circle. By means of the Luttinger theorem,^38−40^ we can extract the amount of charge tunneling from graphene to RuCl_3_, about 4.1 × 10^13^ cm^–2^, consistent with previous, if indirect, experimental and computational observations.^8−10,24,29^ When the spacing between layers is increased with a few h-BN layers, the tunnel barrier thickens, resulting in a decreased charge transfer and therefore a lower p-doping level in graphene (∼1.7 × 10^13^ cm^–2^).

Finally, we can quantify the electric dipole generated at the interface by the charge transfer to the RuCl_3_. It is possible to compute the magnitude of the dipole by measuring the variation of the work function across the different regions of the system. By applying a positive voltage to the sample, the incident LEEM electrons transition from mirror mode, with the electrons reflecting before touching the sample surface, to LEEM mode, where the electrons are scattered from sample surface with a landing energy proportional to the applied sample bias. In LEEM mode, the incident electrons can be accepted into unoccupied bands of the sample surface causing a lower reflected intensity than in the mirror mode. The inflection point of this drop in intensity from mirror mode to LEEM mode can be interpreted as the work function of the sample surface when accounting for the work function of the LEEM cathode.

In Figure 3(a), selected LEEM images collected just below the mirror mode transition display the boundary of the three regions discussed above. The line profiles in Figure 3(b–d) show the average work function across each boundary in the directions indicated by the arrows in the corresponding LEEM images. The profile analysis highlights a difference in the work function of about 230 meV across the interface between Gr/RuCl_3_ and Gr/h-BN. When the h-BN buffer layer is also present, the shift in the work function is reduced by 160 meV. This difference with respect to the Gr/h-BN region agrees with the 70 meV difference between the Gr/h-BN/RuCl_3_ and Gr/RuCl_3_ regions. Previously, Yu and co-workers demonstrated that the work function of graphene can be substantially affected by the dipole formed by surface adsorbates.^32^ Analogously, here we estimate the magnitude of the electric field at the interface knowing the value of the chemical potential and the value of the work function, with respect to pristine graphene. In the presence of a dipole at the interface, the work function of graphene can be written as
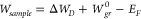
1where Δ*W*_*D*_ is the offset of work function due to the dipole at the interface, *W*_*gr*_^0^ is the intrinsic work function of the undoped graphene, and *E*_*F*_ is the position of the Fermi level.^32^ We can therefore evaluate the magnitude of the electric dipole with respect to the pristine sample simply considering the measured work function difference across the different regions and adding this to the corresponding difference in the *E*_*F*_ position with respect to the Dirac point. This results in an electric dipole energy of ∼1 eV and ∼660 meV for Gr/RuCl_3_ and Gr/h-BN/RuCl_3_ regions, respectively.

**Figure 3:**
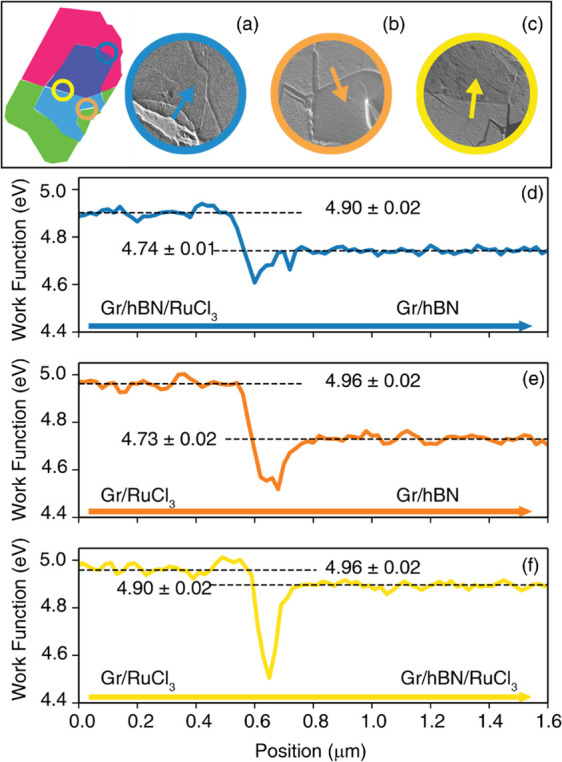
(a–c) Mirror mode LEEM images of selected regions of interest: (a) RuCl_3_-hBN-Gr/Gr, (b) RuCl_3_-Gr/Gr, (c) RuCl_3_-Gr/RuCl_3_-hBN-Gr. (d–f) Surface work function measured across the boundary separating the different regions along the arrows drawn in the microscopy panels.

In conclusion, we used a combination of experimental techniques, including nanoXPS and nanoARPES and LEEM, to investigate the electronic properties of the Gr/RuCl_3_ heterostructure. The results showed direct evidence of significant charge transfer from graphene to RuCl_3_, leading to a doping-induced Mott transition and potential enhancement of the Kitaev physics. LEEM measurement also allowed us to provide an estimate of the dipole moment formed at the interface between RuCl_3_ and graphene, instrumental for comprehensive device modeling. This work lays out valuable insights into the electronic properties of Gr/RuCl_3_ heterostructures and its potential for future applications, where the passive control of the doping level in graphene is at the foundation of low-power electronics and light-harvesting devices.

## Supporting Information Available

Device fabrication methods including atomic force microscopy characterization of the device. Experimental details for nXPS and nARPES experiment including photon energy and light polarization used. LEEM measurements details (PDF)
