# Supplementary material for: Direct Visualization of the Charge Transfer in a Graphene/α-RuCl3 Heterostructure via Angle-Resolved Photoemission Spectroscopy
Source: Nano Lett. 2023 Aug 28;23(17):8000–5. doi: 10.1021/acs.nanolett.3c01974 (PMC10510581; doi:10.1021/acs.nanolett.3c01974)
Supplement: Supplementary file 1 — nl3c01974_si_001.pdf [file nl3c01974_si_001.pdf]

Supporting Information: Direct visualization of the charge transfer in Graphene/α-$RuCl_3$ heterostructure via angle resolved photoemission spectroscopy.

Antonio Rossi[1,2,3], Cameron Johnson[2], Jesse Balgley[4], John C. Thomas[2], Luca Francaviglia[2], Riccardo Dettori[5], Andreas K. Schmid[2], Kenji Watanabe[6], Takashi Taniguchi[7], Matthew Cothrine[8], David G. Mandrus[8], Chris Jozwiak[1], Aaron Bostwick[1], Erik A. Henriksen[4]*, Alexander Weber-Bargioni[2] and Eli Rotenberg[1]*

1-Advanced Light Source, Lawrence Berkeley National Laboratory, Berkeley, California, 94720 USA

2-The Molecular Foundry, Lawrence Berkeley National Laboratory, Berkeley, California, 94720 USA

3- Center for Nanotechnology Innovation @ NEST, Istituto Italiano di Tecnologia, Pisa, 56127 Italy

4- Department of Physics and Institute for Materials Science and Engineering, Washington University in St. Louis, St. Louis, Missouri 63130, United States

5-Physical and Life Sciences Directorate, Lawrence Livermore National Laboratory, Livermore, California 94550, United States

6-Research Center for Functional Materials, National Institute for Materials Science, 1-1 Namiki, Tsukuba, 305-0044 Japan

7-International Center for Materials Nanoarchitectonics, National Institute for Materials Science, 1-1 Namiki, Tsukuba, 305-0044 Japan

8-Material Science & Technology Division, Oak Ridge National Laboratory, Oak Ridge, Tennessee 37831, USA

Device fabrication

Graphene, hexagonal boron nitride (hBN), and $RuCl_3$ flakes were isolated via mechanical exfoliation and atomic force microscopy was used to confirm the flake thicknesses (Fig. S1). A thick h-BN flake is used as a substrate for the device ensuring a flat support surface. The sample was prepared using a dry van der Waals stacking technique[39] to pick up flakes of graphene, thin hBN (~2 nm), $RuCl_3$, and thick hBN (~40 nm), successively, using an adhesive layer of poly(bisphenol A carbonate) (PC). The heterostack was deposited at 180 ˚C onto a prepatterned gold pad to ensure a contact with the ground, which was thermally evaporated on an SiO_2/p-Si substrate.

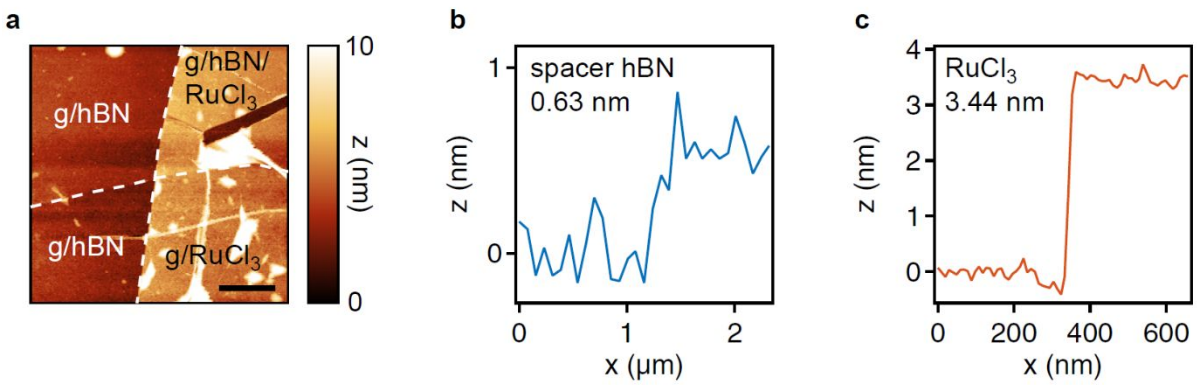

Fig S 1 (a)AFM image of the three regions of interest, scale bar is 500 nm. (b) AFM profile characterization of the h-BN layer sandwiched between graphene and $RuCl_3$. (c) AFM profile characterization of the $RuCl_3$ flake.

Polymer residues are removed by nanobrooming with an atomic force microscope [40]. We first acquired images to identify the area to be cleaned. We used a Park NX10 microscope in non-contact AFM mode with PPP-NCHR probes manufactured by Nanosensors (nominal resonance of 330 kHz, nominal stiffness of $42Nm^{-1}$). We then switched to contact mode for the actual cleaning and scanned the selected area with Arrow-CONTPt probes by NanoWorld (nominal resonance 14 kHz, nominal force constant 0.2 Nm/m). We typically chose values between 65 nN and 100 nN as force setpoints in contact mode and scan rates of 0.5-1 Hz. We used dull tips and assumed a tip radius of about 5nm. We therefore set the distance between two adjacent scanning lines to be equal or shorter than 5nm not to leave dirty gaps.

Experimental details

NanoARPES measurements are performed at beamline 7.0.2 at the Advanced Light Source (ALS) facility at Lawrence Berkeley National Laboratory. The lateral resolution is below 1 um. The nanoARPES data are collected using 150 eV photon energy, while 350 eV excitation is used for core level analysis (nanoXPS). Linear-horizontal polarized light is employed. All the measurements were performed at room temperature.

LEEM measurements were performed on the quantum spin polarized LEEM in the Molecular Foundry at Lawrence Berkeley National Laboratory. LEEM is a UHV electron microscope that uses electron optics to prepare a plane wave of electrons which reflect/scatter from the sample surface with landing energies ranging from 0-500 eV. The reflected/scattered electrons are then collected by imaging optics to project a real or momentum space image of the surface onto a CMOS electron camera.

(1) Wang, L.; Meric, I.; Huang, P. Y.; Gao, Q.; Gao, Y.; Tran, H.; Taniguchi, T.; Watanabe, K.; Campos, L. M.; Muller, D. A.; Guo, J.; Kim, P.; Hone, J.; Shepard, K. L.; Dean, C. R. One-Dimensional Electrical Contact to a Two-Dimensional Material. *Science* **2013**, *342* (6158), 614–617. https://doi.org/10.1126/science.1244358.

(2) Rosenberger, M. R.; Chuang, H.-J.; McCreary, K. M.; Hanbicki, A. T.; Sivaram, S. V.; Jonker, B. T. Nano-"Squeegee" for the Creation of Clean 2D Material Interfaces. *ACS Appl. Mater. Interfaces* Strain. *J. Mater. Chem. C* **2018**, *6* (8), 2019–2025. https://doi.org/10.1039/C7TC05266A.
